# Antidepressant, anxiolytic and procognitive effects of rivastigmine and donepezil in the chronic mild stress model in rats

**DOI:** 10.1007/s00213-016-4206-0

**Published:** 2016-01-15

**Authors:** Mariusz Papp, Piotr Gruca, Magdalena Lason-Tyburkiewicz, Paul Willner

**Affiliations:** Institute of Pharmacology, Polish Academy of Sciences, 12 Smetna Street, 31-343 Krakow, Poland; Department of Psychology, Swansea University, Swansea, UK

**Keywords:** Chronic mild stress, Anhedonia, Object recognition, Cholinesterase inhibitors, Rivastigmine, Donepezil, Memantine, Imipramine, Rat

## Abstract

**Background:**

The treatment of depression in old age is complicated by frequent co-morbidity with cognitive impairment. Anti-dementia drugs have some efficacy to improve cognitive performance and there is an inconsistent literature regarding the effect of such drugs on depressive symptoms. Here, we have investigated whether anti-dementia drugs would have antidepressant-like and pro-cognitive effects in a well-validated animal model of depression and cognitive impairment, chronic mild stress (CMS).

**Methods:**

Rats were subjected to CMS for a total of 8 weeks. After 2 weeks, subgroups of stressed and non-stressed animals were treated daily, for 5 weeks followed by 1 week of drug withdrawal, with vehicle, imipramine (10 mg/kg), rivastigmine (2 mg/kg), donepezil (0.3 mg/kg) or memantine (5 mg/kg). Sucrose intake was tested weekly, and animals were also tested in the elevated plus maze (at week 7) and in an object recognition task (at weeks 7 and 8).

**Results:**

CMS decreased sucrose intake, had an anxiogenic effect in the elevated plus maze, and impaired performance in the object recognition test. Imipramine, rivastigmine and donepezil normalized performance in all three tests. Memantine had anxiolytic and pro-cognitive effects, but did not reverse CMS-induced anhedonia.

**Discussion:**

The fact that all three anti-dementia drugs reversed CMS-induced cognitive impairment and that cholinesterase inhibitors, but not memantine, have antidepressant-like effects in this model suggest that different mechanisms may underlie CMS-induced anhedonia and cognitive impairment. We discuss the clinical implications of these findings.

## Introduction

The treatment of depression has advanced little in the half century since the introduction of antidepressant drugs into clinical practice: while newer drugs generally have an improved side effect profile and some of them have a slightly greater efficacy than older antidepressants, a substantial proportion of patients remain refractory to treatment and the onset of action remains unacceptably slow (Trivedi et al. 2006; Holtzheimer and Mayberg 2011; Belzung 2014). However, recent experience with experimental treatments such as ketamine (Zarate et al. 2006a, b; Diazgranados et al. 2010) and deep brain stimulation (Mayberg 2009; Hamani et al. 2011) provides evidence that the treatment-resistant depression is in principle treatable (Willner et al. 2014).

The treatment of depression in old age is particularly problematic because in addition to their general limitations, antidepressants are less effective in older patients (Kok et al. 2012; Calati et al. 2013). This in part reflects the frequent co-morbidity of old-age depression with cognitive decline (Weisenbach et al. 2012; Aziz and Steffens 2013). In order to address this co-morbidity, a number of studies have been conducted in which anti-dementia drugs were used to augment antidepressant treatment in later life. Positive antidepressant effects of augmentation with acetyl cholinesterase (AChE) inhibitors have been reported (e.g. Cummings et al. 2006; Rozzini et al. 2007; Spalletta et al. 2013) but there are also negative reports (e.g. Holtzheimer et al. 2008; Reynolds et al. 2011) and the overall picture is not encouraging (McDermott and Gray 2012). Another anti-dementia drug memantine, an NMDA receptor antagonist, has generally proved ineffective as an antidepressant (Sani et al. 2012), at least in unipolar depression, when used either as antidepressant augmentation (e.g. Muhonen et al. 2008; Smith et al. 2013; Omranifard et al. 2014) or as monotherapy for late life depression (e.g. Zarate et al. 2006a, b; Lenze et al. 2012).

Despite these discouraging clinical findings, we considered that, since some of the studies of cholinesterase inhibitors did report positive antidepressant effects, it might be worthwhile to ask whether such effects could be detected in a well-validated animal model of depression, since this would enable investigation of underlying mechanisms, leading, potentially, to refinement of the therapeutic approach. We have therefore investigated the effects, in the chronic mild stress model (Willner 1997, 2005), of two acetylcholinesterase (AChE) inhibitors that are in clinical use as anti-dementia drugs, donepezil and rivastigmine. We also studied memantine, which, on the basis of the unequivocal clinical literature, was predicted to be ineffective as an antidepressant. In order to confirm bio-availability of the three drugs, and appropriate dose selection, their effects were also examined in tests of anxiety (the elevated plus-maze) and cognition (novel object recognition). A number of studies have reported that CMS, in addition to its anhedonic effect, also impairs cognitive performance (Orsetti et al. 2007; Elizalde et al. 2008; Briones et al. 2012; Solas et al. 2013). However, other than one small and uninterpretable study of memantine (Quan et al. 2011), and a second study in which donepezil improved memory to a similar extent in CMS and control mice (Maratha and Mahadevan 2012), we have been unable to identify previous studies in which the ability of anti-dementia agents to reverse these deficits was investigated. Therefore, this study also served to investigate whether donepezil, rivastigmine and memantine would reverse a CMS-induced cognitive impairment. The tricyclic antidepressant imipramine was also tested, as a positive control.

## Methods

### Subjects

A total of 96 male Wistar rats (Charles River, Germany) were brought into the laboratory 1 month before the start of the experiment. The animals were 3 months old at the start of the stress procedure and 5 months old at the end. Body weights were measured at four time points during the course of the experiment (see below). Except as described below, the animals were singly housed with food and water freely available, and were maintained on a 12-h light/dark cycle (lights on at 08.00 h) under conditions of constant temperature (22 ± 2 °C) and humidity (50 ± 5 %). Behavioural experiments were conducted between 09.00 and 15.00 h. All procedures used in this study were conducted in compliance with the rules and principles of the 86/609/EEC Directive, and were approved by the Bioethical Committee of the Institute of Pharmacology, Polish Academy of Sciences, Krakow, Poland.

### Chronic mild stress (CMS) procedure

**Fig. 1:**
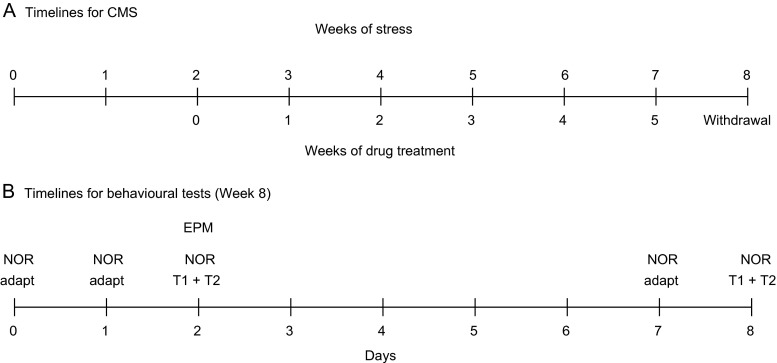
Experimental timelines. **a** Timelines for CMS. Before the onset of CMS (week 0), animals were adapted for 2 weeks to laboratory and housing conditions followed by 7 weeks of baseline sucrose consumption tests. CMS (or no stress for the control group) was administered for 8 weeks, with drug treatment (saline, rivastigmine, donepezil, memantine or imipramine) administered daily for 5 weeks, beginning after 2 weeks of CMS, followed by a week of drug withdrawal. Sucrose consumption tests were conducted weekly, from 10.00 to 11.00 on Tuesdays. **b** Timelines for behavioural tests, conducted in the final week, following withdrawal after the 5 weeks of drug treatment. The first adaptation to the novel object recognition (*NOR*) apparatus (NOR adapt) took place 1 day before last drug dose. NOR trials (T1 and T2) and elevated plus maze (*EPM*) tests took place on the days shown. CMS continued throughout the entire period: on the night before behavioural tests, animals received tilting or no stress; after the tests, scheduled stressors were applied. See text for further details

Following 2 weeks of adaptation to laboratory and housing conditions, animals were adapted to consumption of a 1 % sucrose solution in 7 weekly 1-h baseline tests following 14 h food and water deprivation. Singly housed animals in their home cages were presented with 200 ml of sucrose in a 300 ml polythene bottle equipped with a rubber bung and a stainless steel ball-tip nozzle (North Kent Plastics, UK). Fresh sucrose solutions were made up for each test; following the test, bottles were washed using warm tap water and no detergent. Sucrose intake was calculated by weighing bottles before and after the test (Fig. 1).

On the basis of their sucrose intakes in the final baseline test, the animals were divided into two matched groups: control (CON) and to-be-stressed (STR). Control animals were housed in separate rooms and had no contact with the stressed animals; they were deprived of food and water for 14 h preceding each sucrose test, but otherwise, food and water were freely available in their home cage. Sucrose tests were administered for 1 h weekly, from 10.00 to 11.00 h on Tuesdays; other than food/water deprivation, no stressors were administered in the previous 24 h. Stress was administered for a total of 8 weeks, and consisted of the following: two periods of food or water deprivation, two periods of 45° cage tilt, two periods of intermittent illumination (lights on and off every 2 h), two periods of soiled cage (250 ml water in sawdust bedding), one period of paired housing, two periods of low intensity stroboscopic illumination (150 flashes/min), and three periods of no stress. All stressors were 10–14 h of duration and were applied individually and continuously, day and night.

On the basis of their sucrose intakes following 2 weeks of stress, both CON and STR groups were divided further into matched subgroups (*n* = 8 per group), which for the next 5 weeks received once-daily intraperitoneal administration of vehicle (saline, 1 ml/kg), imipramine HCl (10 mg/kg), rivastigmine (2 mg/kg), donepezil (0.3 mg/kg) or memantine (5 mg/kg). The drugs were administered at approximately 10.00 h and the weekly sucrose tests were carried out 24 h following the previous drug injections. After 5 weeks, all drug treatments were terminated and 24 h later, the animals were individually removed from their housing rooms to another room for testing in elevated plus maze and novel object recognition tests (see below). At least 15 min was allowed for habituation to the new environment before the behavioural test. After completing these tests, the animals were returned to their housing rooms and the CMS procedure was continue for a further week. After 1 week of drug withdrawal, the novel object recognition test was repeated. Stress was continued throughout the entire period of drug treatment and withdrawal. On the night before behavioural tests, animals received tilting or no stress; after the tests, scheduled stressors were applied (Fig. 1).

The study was run as two replications, the first including saline (SAL), imipramine (IMI) and rivastigmine (RIV) groups, and the second including SAL, donepezil (DON) and memantine (MEM) groups.

### Behavioural tests

#### Elevated plus maze (EPM)

The animals were tested in non-transparent apparatus, which consisted of two open (50 × 11 cm) and two closed (50 × 11 × 40 cm) arms. The apparatus was elevated 50 cm above the floor and was illuminated by two 25 W bulbs located beneath the open arms. The animal was individually placed in the centre of the apparatus, and the number of entries into open and closed arms was manually recorded during a 5-min test. An arm entry was defined as a rat having entered an arm with all four legs.

#### Novel object recognition (NOR)

The animals were tested in non-transparent open field (100 cm in diameter, 35 cm high, with the floor divided into painted 16-cm squares). After 10-min adaptation sessions on two successive days, the animals were allowed to explore two identical objects (white cylinders, 7 cm in diameter, 11 cm high) for the time required to complete 15 s of exploration of both objects (T1 session). In a retention trial conducted 1 h later (T2 session), one of the objects presented previously was replaced by a novel object (black prism, 5 cm wide, 14 cm high). Rats were returned to the open field for 5 min and the duration of exploration of each object (i.e. sitting in close proximity to the objects, sniffing or touching them) was measured by a trained observer who was blind to drug treatment. A NOR index was calculated according to the following formula: time of novel object exploration divided by time of novel plus familiar object exploration, multiplied by 100. During NOR sessions, the number of line crossings was recorded as a measure of locomotor activity.

#### Drugs

Drugs used were imipramine HCl (RBI, Natic, MA, USA), rivastigmine, donepezil and memantine (Sequoia Research Products Ltd, Pangbourne, UK). All drugs were dissolved in 0.9 % sterile saline, which was used as a vehicle control treatment. The doses were selected on the basis of data in the literature and our preliminary experiments showing their efficacy in the behavioural assays used in this study. Doses were calculated as the salts, and all injections were made in a volume of 1 ml/kg of body weight.

#### Statistical analysis

Initial analyses confirmed that the two saline groups did not differ significantly on any parameter. Therefore, these two groups were combined for the purposes of analysis and (with one exception) display. Data were analysed by analysis of variance (anova) with the between-groups factors stress (CON vs. STR) and drug (SAL, IMI, RIV, DON, MEM). In some analyses, a further within-subjects factor was included, as described below. Significant interactions were followed up with lower-order anovas and Bonferroni tests, or *t* tests, as appropriate.

## Results

### Body weight

Animals were weighed at four time points, the start of the stress procedure (week 0: overall mean body weight = 330 g), after 2 weeks of CMS (week 2), after 5 weeks of CMS plus drug treatment (week 7), and after a further week of CMS during drug withdrawal (week 8). Anova showed a significant effect of drug [F(4,86) = 3.05, *p* < 0.05] and significant drug × weeks [F(12,258) = 26.8, *p* < 0.001] and stress × weeks [F(3258) = 8.02, *p* < 0.001] interactions, while the main effect of stress and the stress × drug and stress × drug × weeks interactions were nonsignificant [all Fs <1]. Further analysis showed that STR animals were a little heavier than CON at baseline but gained less weight than CON during the first 2 weeks of stress (mean weight gain 13 vs. 24 g), such that that the two groups were very similar in body weight (within 3 g) thereafter, and that during the 5-week period of drug treatment, the SAL and DON groups each gained a mean of 28 g, the RIV and MEM groups gained significantly less weight (mean of 12 g), while the IMI group lost 16 g.

### Sucrose intake

**Fig. 2:**
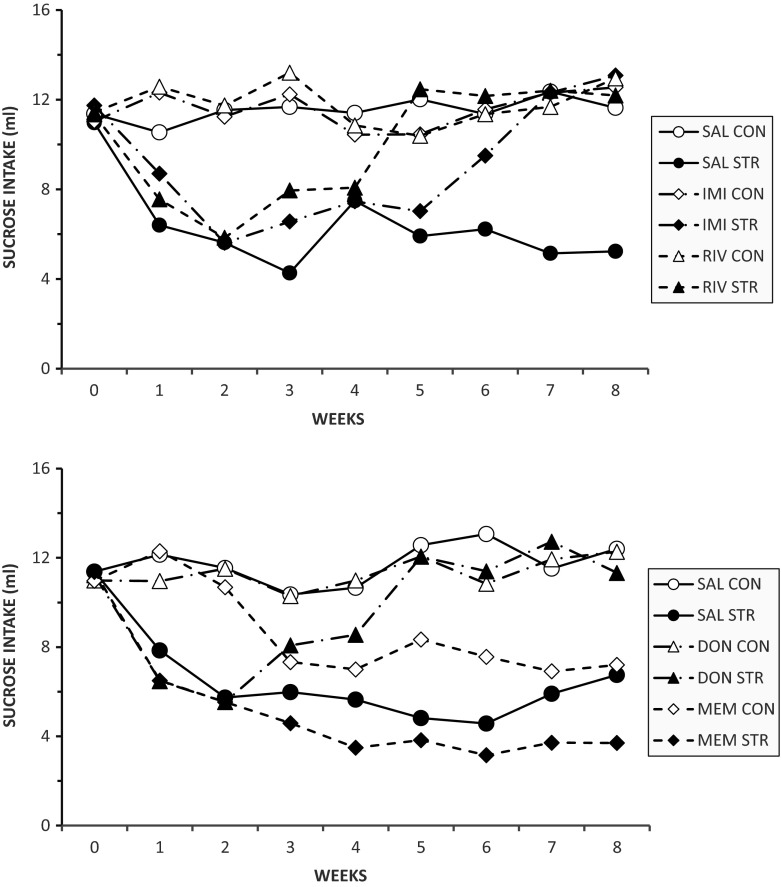
Sucrose intake in 60-min weekly tests by non-stressed control groups (*CON*) or animals exposed for 8 weeks to chronic mild stress (*CMS*). Drugs were administered for 5 weeks (weeks 3–7). The *upper panel* shows effects of saline (*SAL*), imipramine (*IMI*) and rivastigmine (*RIV*); the *lower panel* shows effects of saline (*SAL*), donepezil (*DON*) and memantine (*MEM*). Values are group means. Error bars and significance indicators are omitted for clarity

Sucrose intakes are shown in Fig. 2, which for clarity shows the results of the two experiments separately. Anova confirmed a significant stress × drug × weeks interaction [F(32,688) = 3.15, *p* < 0.001]. This results from the following effects:

(i) All STR groups showed a decrease in sucrose intake during weeks 1 and 2. In a further analysis, sucrose intake was expressed as a function of body weight (g sucrose/kg body weight). Anova confirmed that sucrose intake was also significantly lower at week 2 when expressed in this way [stress × weeks interaction: F(1,94) = 117.3, *p* < 0.001; results not shown].
(ii) All CON groups maintained a steady high level of sucrose intake throughout the study, with the exception of the CON-MEM group, for which intake fell during the first week of drug treatment (week 3) and remained at roughly the same level thereafter.
(iii) The STR-IMI, STR-RIV and STR-DON groups all recovered to control levels of intake. This occurred at week 3 of drug treatment (i.e. week 5 of CMS) in the RIV and DON groups and week 5 of drug treatment (i.e. week 7 of CMS) in the IMI group.
(iv) The STR-MEM group did not recover. A separate Anova confined to the two MEM groups for the period weeks 3–6 confirmed that the effect of stress remained significant despite the decreased intake in the CON group [F(1,14) = 11.56, *p* < 0.001] and there was no significant stress × weeks interaction [F(5,70) = 0.4].

### Elevated plus-maze

**Fig. 3:**
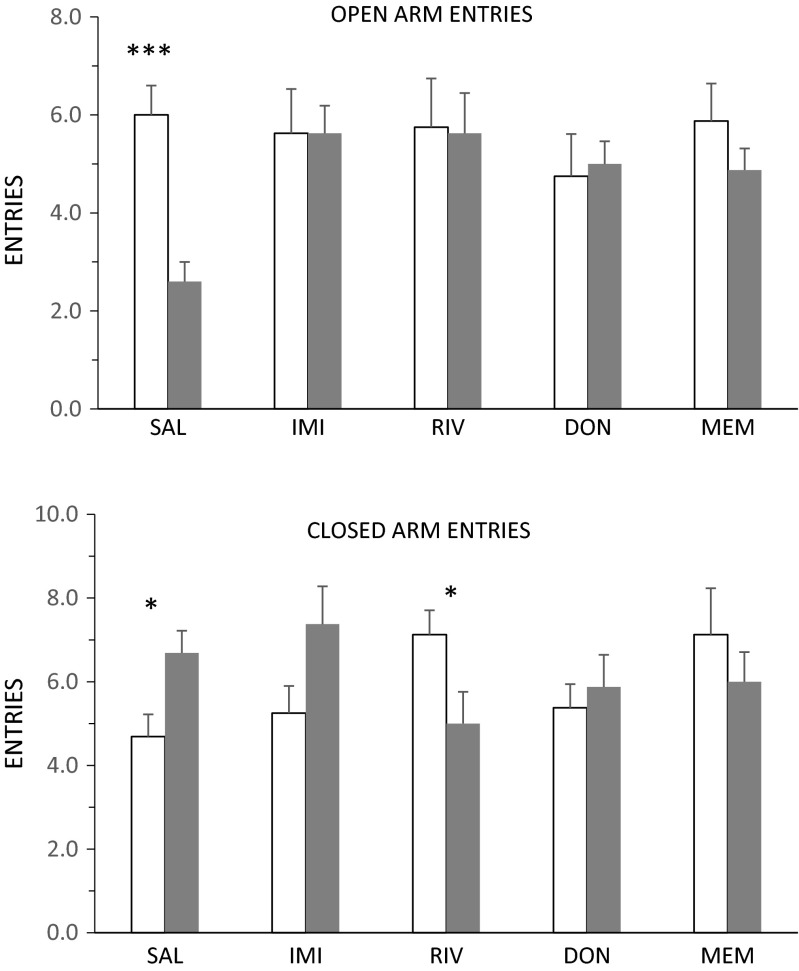
Number of entries to the open (*upper panel*) and closed (*lower panel*) arms of the elevated plus maze in non-stressed animals (*white bars*) or animals subjected for 7 weeks to CMS (*grey bars*). Both groups received daily administration, for 5 weeks, of saline (*SAL*), imipramine (*IMI*), rivastigmine (*RIV*), donepezil (*DON*) or memantine (*MEM*). Values are means + standard error. *p < 0.05; ***p < 0.001, control vs. CMS

In the EPM, there was a pronounced decrease in open arm entries in the STR-SAL group relative to the STR-CON group (Fig. 3, upper panel: t(30) = 5.12, *p* < 0.001), but there was no significant effect of stress in any of the drug-treated groups: that is, the effect of stress was reversed by all four drug treatments [stress × drug interaction: F(1,86) = 3.60, *p* < 0.01]. Results were similar for open arm time (not shown). There were some smaller effects on closed arm entries with no clear pattern [Fig. 3, lower panel; drug × stress interaction: F(4,86) = 3.70, *p* < 0.01]. Total entries did not differ significantly between groups.

### Novel object recognition

**Fig. 4:**
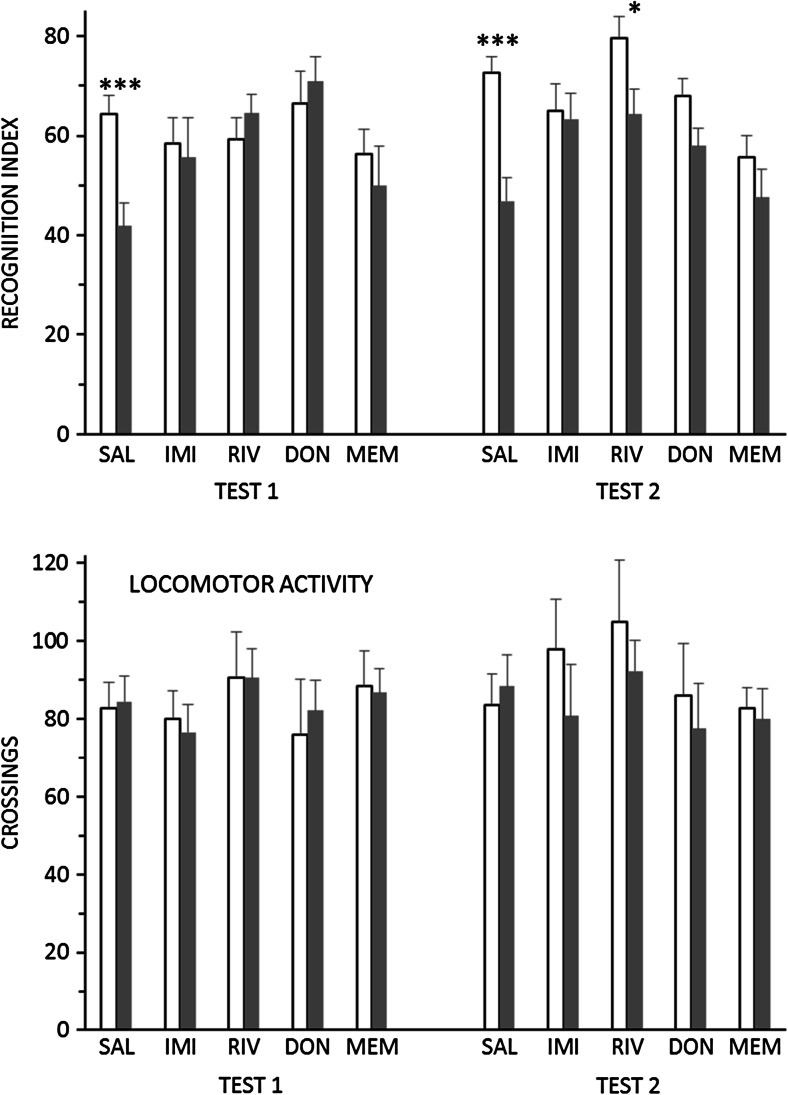
Behaviour in the novel object recognition (NOR) test by non-stressed animals (*white bars*) or animals subjected for 7 and 8 weeks to CMS (*grey bars*). *Upper panel*: NOR index (%); *lower panel*: number of lines crossed. Both groups received daily administration of saline (*SAL*), imipramine (*IMI*), rivastigmine (*RIV*), donepezil (*DON*) or memantine (*MEM*). Test 1 followed 5 weeks of drug administration; test 2 followed 1 week of drug withdrawal. Values are means + standard error. *p < 0.05; ***p < 0.001, control vs. CMS

In the NOR test (Fig. 4, upper panels), SAL-treated stressed animals showed a marked decrease, relative to SAL-treated controls, in exploration of the novel object, which was highly significant in both tests [t(1,30) = 3.85 and 4.47, *p* < 0.001]. Behaviour was normalized by all four drug treatments [stress × drug interaction: F(4,86) = 3.40, *p* < 0.02]. The stress × drug × tests interaction was nonsignificant [F(4,86) = 1.1], indicating that stress × drug interactions were similar in the two tests. However, visual inspection suggests that the effect of RIV to reverse the effect of stress was present in the first test but not the second, and a further post hoc test confirmed that the difference between CON and STR RIV-treated animals was marginally significant in test 2 [t(1,14) = 2.30, *p* < 0.05].

Locomotor activity was not significantly affected by stress or drugs in either test [Fig. 4, lower panels; max F-value for main effects and stress × drug interactions = 1.16].

## Discussion

Chronic mild stress caused a typical anhedonic effect in the sucrose intake test Willner 1997, 2005), as well as an anxiogenic effect in the elevated plus maze and cognitive impairment in the object recognition test. All of these effects were reversed by the prototypical antidepressant drug imipramine. The interpretation of a decrease in sucrose intake as anhedonia has been extensively discussed, and is supported by the demonstration that this effect is independent of effects of stress on body weight (as confirmed here), by CMS-induced impairments in other tests of rewarded behaviour such as place conditioning and brain stimulation reward, and by the ineffectiveness of anxiolytic drugs such as benzodiazepines to reverse these impairments (Willner 1997, 2005).

All three anti-dementia drugs were effective in reversing CMS effects in the open field and object recognition tests. However, while the anticholinesterases rivastigmine and donepezil also reversed stress-induced anhedonia (and so were comparable to imipramine in their overall effects), the NMDA antagonist memantine did not. The onset of action of donepezil and rivastigmine was somewhat faster than that of imipramine (recovery in 3 vs. 5 weeks) but these differences may be dose-related. A limitation of the present study is that it was conducted in young animals. This was a deliberate choice, to enable a clean evaluation of the interaction between drug effects and CMS while avoiding complications arising from age-associated cognitive decline. However, as anti-dementia drugs are typically prescribed to older patients, a replication of the study in older animals would be warranted.

Anxiogenic effects, while not universally observed, have frequently been described in animals subjected to chronic mild stress, and, like anhedonic effects, are reversed by chronic antidepressant treatment (e.g. Surget et al. 2008; Farley et al. 2010; Wang et al. 2014). We are unaware of any previous studies of the effects of anticholinesterases or NMDA antagonists on anxiety-like behaviour in the CMS model. In normal animals, a recent study found that chronic administration of fluoxetine increased hippocampal AChE activity, while knockdown of AChE in the hippocampus increased anxiety-like behaviour in the elevated plus-maze (Mineur et al. 2013), suggesting an anxiogenic effect of hippocampal AChE inhibition. However, anxiolytic-like effects of systemically administered AChE inhibitors have also been reported (e.g. Cutuli et al. 2008; Chen et al. 2011; Zarrindast et al. 2011). Results with memantine are similarly mixed, with some studies reporting anxiolytic-like effects (Bertoglio and Carobrez 2003; Minkeviciene et al. 2008) and others finding memantine to be ineffective as an anxiolytic (Karcz-Kubicha et al. 1997; Kotlinska and Bochenski 2008; Koltunowska et al. 2013). As noted in the Introduction, in the present study, the purpose of testing animals in the elevated plus-maze was primarily to confirm that drugs were administered at behaviourally active doses. Further studies of the potential anxiolytic activity of these drugs in the CMS model and other well-validated models of depression could provide a clearer picture of their potential relevance to mixed anxiety-depression conditions.

The impairment of object recognition performance by CMS, and its remediation by chronic antidepressant treatment, is consistent with earlier findings (Orsetti et al. 2007; Elizalde et al. 2008; Briones et al. 2012; Solas et al. 2013). In aged animals, CMS also increases biochemical markers associated with Alzheimer’s disease (El-faramawy et al. 2009; Briones et al. 2012; Solas et al. 2013; Yang et al. 2014), indicating that, in addition to its utility as an animal model of depression, the CMS procedure may have value as a model to investigate the early stages of dementia (e.g. Cuadrado-Tejedor et al. 2012; Lisowski et al. 2013). Our observation that imipramine, rivastigmine and donepezil reversed both CMS-induced anhedonia and cognitive impairment, while memantine reversed only the cognitive impairment, suggests the possibility that different mechanisms may mediate these two behavioural effects of CMS. Indeed, in the final set of tests, following a week of drug withdrawal, there was a suggestion of a double dissociation: memantine reversed the impairment of object recognition and rivastigmine reversed anhedonia, but not vice versa. (However, the lack of effect of rivastigmine results from an increase in the discrimination index in the control group (Fig. 4) and so may not be reliable.) While there is a substantial literature describing the mechanisms involved in CMS-induced anhedonia and its reversal by antidepressant drugs (reviewed by Willner et al. 2013), the mechanisms underlying CMS-induced cognitive impairment remain to be elucidated.

The absence of an antidepressant-like effect of memantine in the present study, in the context of evidence of behavioural activity of memantine in other tests, is consistent with clinical evidence that memantine is not an effective antidepressant (Sani et al. 2012). Memantine is different in this respect from ketamine, for which antidepressant properties are well established: the explanation may be that while both drugs are NMDA receptor antagonists, they differ in the effect on glutamatergic neurotransmission and downstream intracellular signaling: for example, ketamine, but not memantine, increases hippocampal BDNF expression (Gideons et al. 2014). In contrast to the present results (and the clinical outcomes), two previous studies have reported positive antidepressant-like effects of memantine in the CMS model (Quan et al. 2011; Réus et al. 2012). The discrepancy may be dose-related: the earlier studies used a considerably higher dose of memantine (20 vs. 5 mg/kg) that is associated with adverse side effects (Creeley et al. 2006, 2008).

As noted earlier, there is a mixed clinical picture regarding potential antidepressant effects of AChE inhibitors (see Introduction). However, the fact that there are any positive reports could be considered surprising, in the context of an extensive earlier literature reporting that anticholinesterases exacerbate depression (Janowsky et al. 1974; Janowsky and Risch 1984). The variable clinical outcomes may reflect the fact that, by increasing synaptic levels of acetylcholine, AChE inhibitors increase stimulation of both muscarinic and nicotinic acetylcholine receptors, and these two receptors may be differentially involved in depression. Muscarinic receptor antagonists appear to have antidepressant properties: there is an extensive older literature describing antidepressant effects of atropine (Hoch and Mauss 1932; Janowsky and Risch 1984), and a more recent literature reporting similar effects of scopolamine (Furey and Drevets 2006; Hasselmann 2014). Consistent with the clinical literature, atropine (Papp et al. 1996) and scopolamine (Geoffroy et al. 1990) have also been found to exert antidepressant-like effects in the CMS and learned helplessness models, respectively. Conversely, antidepressant-like effects in these two models have been reported for nicotinic receptors agonists (Ferguson et al. 2000; Andreasen et al. 2011), and there is current interest in the clinical potential of nicotinic agonists as antidepressants (Philip et al. 2010; Zurkovsky et al. 2013). Thus, it is likely that the antidepressant-like effects of donepezil and rivastigmine that were observed in the present study would be blocked by nicotinic, but not muscarinic, antagonists; further mechanistic studies are needed to test this prediction. More generally, it seems likely that the outcome of anticholinesterase treatment of depression may depend on the balance of stimulation at muscarinic and nicotinic receptors, which might vary as a function of drug dose, mood status, and baseline level of cholinergic activity. Further investigation is needed to elucidate these relationships.

A single controlled trial of donepezil found no benefit of adding donepezil to antidepressant maintenance for older adults with normal cognition or mild cognitive impairment (Reynolds et al. 2011). However, positive antidepressant effects in patients with Alzheimer’s disease have been reported in two open trials of a rivastigmine transdermal patch (Spalletta et al. 2013, 2014) and a controlled trial of oral rivastigmine (Mowla et al. 2007). Consistent with these clinical data and the present observations, chronic administration of rivastigmine also had characteristic antidepressant-like and anxiolytic effects in olfactory bulbectomized animals (Islam et al. 2014). Rivastigmine also restored hippocampal neurogenesis and associated enzyme activities in bulbectomized animals; and all of these effects were blocked by co-treatment with a 5HT1A receptor antagonist (Islam et al. 2014). Destruction of cholinergic neurons is known to decrease hippocampal 5HT levels (Nakamura et al. 1992), suggesting that an adequate level of cholinergic activity is necessary for normal activity at hippocampal 5HT terminals, and that rivastigmine may enhance hippocampal 5HT activity in a manner comparable to conventional antidepressants (Willner et al. 2013). Alongside these earlier data, the present results provide grounds for cautious optimism that there may be a place for AChE inibitors in the treatment of depression, though realistically, this would in practice be restricted to older patients experiencing, or at risk of, cognitive decline.
